# Nucleic acid amplification techniques for rapid diagnosis of nontuberculous mycobacteria: A protocol of systematic review and meta-analysis

**DOI:** 10.1371/journal.pone.0250470

**Published:** 2021-04-22

**Authors:** Guocan Yu, Yanqin Shen, Xudong Xu, Lihua Lin

**Affiliations:** Zhejiang Tuberculosis Diagnosis and Treatment Center, Zhejiang Chinese Medicine and Western Medicine Integrated Hospital, Hangzhou, Zhejiang, 310003, China; The University of Georgia, UNITED STATES

## Abstract

**Background:**

Nontuberculous mycobacteria (NTM) infection is similar to *Mycobacterium tuberculosis* (MTB) infection. Early clinical identification and differentiation of NTM and MTB infections continues to be a major challenge. Nucleic acid amplification tests (NAATs) have the ability to efficiently and rapidly detect pathogens and are widely used in mycobacterial infections. The objective of this study is to estimate the diagnostic accuracy of NAATs for NTM.

**Methods:**

We will search candidate studies that assessing the accuracy of NAATs for diagnosis of NTM through PubMed, Embase and the Cochrane Library until May 2021. Studies with full text that meet the inclusion criteria will be included. Following a revised tool for Quality Assessment of Diagnostic Accuracy Studies (QUADAS-2), two researchers will independently evaluate the study quality. The STATA software (version 15.0) will be used to carry out meta-analyses. When heterogeneity is observed, subgroup analyses and meta-regression analyses will be used to explore sources of heterogeneity. Sensitivity analyses will be used to check the robustness of analyses.

**Conclusion:**

We hope that this study will provide meaningful evidence for the early and rapid diagnosis of NAATs for NTM, which will help to guide the treatment of NTM and improve the prognosis of patients.

## Introduction

The genus Mycobacterium includes three species, *Mycobacterium tuberculosis* (MTB), *Mycobacterium leprae*, and nontuberculous mycobacteria (NTM) [1]. NTM is widespread in the environment and is also considered to be an environmental *Mycobacterium* [2]. However, NTM can also cause a number of infections, of which pulmonary infections are the most common, accounting for 65–90% [3]. The incidence of NTM infections is on the rise worldwide, and the pathogen is receiving increasing attention [4]. Approximately 200 NTM species have been identified to date, of which at least 60 NTM species are considered to be the causative pathogens of human *Mycobacterium* infection. The treatment regimens for infections caused by different types of NTM are different [5], therefore, early identification of the type of NTM is crucial for treatment. Acid-fast bacilli (AFB) smear does not possess the ability to differentiate between NTM and MTB infections. Conventional diagnosis of NTM requires the results of *Mycobacterium* culture before species identification, and *Mycobacterium* culture takes several weeks and sensitivity is still at the low side [6]. *Mycobacterium* culture does not comply with the need for early and rapid diagnosis for NTM. On the other hand, the clinical symptoms of NTM and MTB infections are very similar, and NTM infections can easily be misdiagnosed as MTB infections, resulting in inappropriate or delayed treatment [7]. Early clinical identification and differentiation between NTM and MTB infections continue to be a major challenge [8]. Missed or misdiagnosed NTM infections can lead to serious complications such as destruction of the lung, which can affect patient prognosis [9]. Therefore, rapid diagnosis of NTM and differentiation of NTM species is essential in the management of NTM infection.

Nucleic acid amplification tests (NAATs) are increasingly used in infectious diseases [10]. NAATs have the ability to rapidly detect pathogens and are widely used in *Mycobacterium* infections [11,12]. Several studies have shown that NAATs also have good diagnostic accuracy in the early diagnosis of NTM [13,14]. Early and rapid diagnosis of NTM by NAATs and identification of the species, which can effectively guide treatment and thus improve the prognosis of patients. However, there is still no systematic review and meta-analysis of NAATs for the diagnosis of NTM. The aim of this study is to comprehensively assess the diagnostic accuracy of NAATs for early and rapid diagnosis of NTM.

## 2. Methods

### 2.1 Design and registration

We will conduct a systematic review and meta-analysis of diagnostic test accuracy. The registration number of this protocol is INPLASY2020110076 on the International Platform of Registered Systematic Review and Meta-Analysis Protocols (INPLASY) [15]. We will follow the Preferred Reporting Items for Systematic Reviews and Meta-Analysis for Diagnostic Test Accuracy (PRISMA-DTA) guideline to report this study [16]. No ethical approval is required for meta-analysis.

### 2.2. Information sources

We will search candidate studies that assessing the accuracy of NAATs for diagnosis of NTM through PubMed, Embase and the Cochrane Library until May 2021. References cited in a review or meta-analysis will also be evaluated to determine additional studies.

### 2.3. Search strategy

GCY and YQS will designed and implemented the comprehensive search strategies. Use of English in the search process. Search strategy of PubMed will be listed as follows:

#1 "Nontuberculous Mycobacteria"[Mesh] OR “Non-Tuberculous Mycobacteria” OR “Atypical Mycobacterium” OR “Nontuberculous Mycobacterium” OR “Mycobacteria, Atypical” OR “Tuberculoid Bacillus” OR “Mycobacterium, Atypical” OR “Atypical Mycobacteria” OR “Mycobacterium terrae” OR “Mycobacterium duvalii” OR “Mycobacterium obuense” OR “Mycobacterium gilvum” OR “Mycobacterium gordonae” OR “Mycobacterium szulgai” OR “Mycobacterium flavescens”
#2 "Mycobacterium"[Mesh] OR Mycobacteria
#3 #1 OR #2
#4 "Nucleic Acid Amplification Techniques"[Mesh] OR "Polymerase Chain Reaction"[Mesh] OR "Real-Time Polymerase Chain Reaction"[Mesh] OR "Reverse Transcriptase Polymerase Chain Reaction"[Mesh] OR "Multiplex Polymerase Chain Reaction"[Mesh] OR "LAMP assay" [Supplementary Concept] OR "loop-mediated isothermal amplification"
#5 #3 AND #4

The other two databases will use a similar search strategy.

### 2.4. Eligibility criteria

#### 2.4.1. Type of studies

Any type of study design such as retrospective studies, prospective studies, case-control studies, if the study had assessed the accuracy of NAATs in diagnosing NTM. True positive (TP), false positive (FP), false negative (FN), and true negative (TN) values for the index tests could be extracted directly or calculated from the original studies. Studies that report only sensitivity or specificity, studies reported in languages other than English, conference abstracts without full articles, and case reports will be excluded.

#### 2.4.2. Participants

Participants of any ethnicity, sex, or age, suspected of having NTM will be included.

#### 2.4.3. Index tests

NAATs (such as polymerase chain reaction [PCR] based techniques and loop-mediated isothermal amplification [LAMP]) are the index tests.

#### 2.4.4. Target conditions

NTM is the target condition.

#### 2.4.5. Reference standard

NTM culture will be described as the reference standard. A composite reference standard does not apply to NTM infection.

#### 2.4.6. Outcomes

The sensitivity and specificity of the index tests will be considered as the main outcome. Sensitivity is the probability that the index tests will detect positive in an infected patient. Specificity is the probability that the index tests will detect negative in a noninfected patient [17].

### 2.5. Literature screening and selection

We will use ENDNOTE X9.2 literature management software to manage related literature. The search candidate studies will be imported into the software. Original studies that diagnosed NTM by the reference standard and have assessed the diagnostic accuracy of NAATs for NTM with full text will be included in this systematic review and meta-analysis. Clear reference standard in the original study.

Two independent researchers (GCY and YQS) will evaluate the candidate studies for inclusion based on inclusion criteria. They will review the title and abstract first, then the full text. If there is disagreement between the two researchers, they will consult with a third investigator (LHL).

### 2.6. Data extraction

We will extract information for each study such as first author; publication date; country; type of study design; patient selection method (such as consecutive and random); TP, FP, FN, and TN values for the index tests; index test type (such as PCR and LAMP); NTM type; specimen types (such as sputum and bronchoalveolar lavage fluid); specimen condition; specimen processing method (e.g., homogenization) and DNA extraction method. As with the literature screening process, the same two researchers will perform the data extraction independently, and if there is any disagreement, it will be handled in the same way as in the literature screening phase.

### 2.7. Methodological quality assessment

Using the revised tool for Quality Assessment of Diagnostic Accuracy Studies (QUADAS-2) [18], two researchers will independently evaluate the risk of bias and applicability of the included studies. The discrepancy will be dealt with in the same way as in the data extraction phase.

### 2.8. Data synthesis and statistical analysis

TP, FP, FN, and TN values for the index tests in each included study will be obtained first, then the estimated pooled sensitivity and specificity of NAAT associated with the 95% confidence interval (CI) will be calculated using bivariate random-effects models. The heterogeneity will be investigated by visual examination of forest plots [19]. When heterogeneity is observed, subgroup analyses and meta-regression analyses will be used to explore sources of heterogeneity. Subgroup analyses and meta-regression analyses will perform on different types of study design; patient selection methods (such as consecutive and random); index test types (such as PCR and LAMP); NTM types; specimen types (such as sputum and bronchoalveolar lavage fluid); specimen conditions; specimen processing methods (e.g., homogenization) and DNA extraction methods. Sensitivity analyses will be used to check the robustness of analyses. At least 4 eligible studies will be required to perform the meta-analysis and subgroup analyses for predefined variable parameters using Stata version 15.0 (Stata Corp., College Station, TX, USA) with the *midas* command [20], and meta-regression analyses using *meqrlogit* command.

### 2.9 Evidence evaluation

We will evaluate all the strength of the body of evidence according to The Grading of Recommendations Assessment, Development and Evaluation (GRADE) guideline [19]. The quality of evidence will be classified into 4 levels: high, moderate, low, and very low. We will assess the factors that determine and can decrease quality of evidence (such as study design, risk of bias, outcomes, patient populations, diagnostic test, important inconsistency in study results, imprecise evidence and high probability of publication bias) to determine the quality of evidence [21].

## 3. Discussion

NTM infection is easily missed or misdiagnosed, and there is an urgent need to strengthen the diagnostic accuracy of NTM. There is no published systematic review and meta-analysis evaluating the accuracy of NAATs for NTM diagnosis to the best of our knowledge. We hope that this study will provide meaningful evidence for the early and rapid diagnosis of NAATs for NTM, which will help to guide the treatment of NTM and improve the prognosis of patients.

## Supporting information

S1 Checklist Preferred Reporting Items for Systematic review and Meta-Analysis Protocols (PRISMA-P checklist). (DOC)

S1 File QUADAS-2 adaptation for this study. (DOCX)
