# Supplementary material for: Nucleic acid amplification techniques for rapid diagnosis of nontuberculous mycobacteria: A protocol of systematic review and meta-analysis
Source: PLoS One. 2021 Apr 22;16(4):e0250470. doi: 10.1371/journal.pone.0250470 (PMC8062022; doi:10.1371/journal.pone.0250470)
Supplement: S1 File — (DOCX) [file pone.0250470.s002.docx]

QUADAS adaptation for this review

Patient selection

1. Was a consecutive or random sample of patients enrolled?: all patients defined for inclusion included consecutively
2. Was a case-control design avoided?
3. Did the study avoid inappropriate exclusions? Were all samples collected analyzed?
   - Patient selection risk of bias: High risk if any of #1-3 was "no"; low risk if all "yes" or inappropriate exclusions unclear; otherwise unclear risk of bias
   - Patient applicability: High risk if target condition not defined; low risk if type of NTM defined; unclear if not described

Index test

1. Were the index test results interpreted without knowledge of the results of the reference standard?
2. If a threshold was used (for index test), was it pre-specified?
   - Index test risk of bias: High risk whenever #4 was "no" or when #4 was unclear and #5 was "no"; low risk when both "yes" or #4 was "yes" and #5 was "unclear"; otherwise unclear risk of bias.
   - Index test applicability: High risk if the index test was evaluated on different specimens that were not separated (sputum, bronchoalveolar lavage) or the specimen types were not defined; low risk if a single specimen type.

Reference standard

1. Is the reference standard likely to correctly classify the target condition?

"Yes" if using NTM culture findings; "No" if not using NTM culture findings; "unclear" otherwise.

1. Were the reference standard results interpreted without knowledge of the results of the index test?
   - Reference standard risk of bias: Low risk if both #6 and #7 "yes"; High risk if both "no"; otherwise unclear risk of bias
   - Reference standard applicability: High risk if the reference standard was not NTM culture; low risk if NTM culture used

Flow and timing

1. Was there an appropriate interval between index test(s) and reference standard? "Yes" if both were taken and analyzed together; "No" if taken on different occasions; "unclear if taken together but analyzed at different times
2. Did all patients receive a reference standard? 100% of samples analyzed for the index test received the reference standard
3. Did patients receive the same reference standard?
4. Were all patients included in the analysis?
   - Flow and time risk of bias: High risk if any of #8-#11 "no"; low risk if all "yes", or #8 unclear and #9-#10 "yes"; otherwise unclear risk of bias
